# Harnessing population genetics and animal models to uncover the genetics of skin scarring

**DOI:** 10.1016/j.bjps.2025.11.010

**Published:** 2025-11-12

**Authors:** Oscar A. Peña, Marisa Cañadas-Garre, Iona Collins, Nicholas J. Timpson, Paul Martin

**Affiliations:** a School of Biochemistry, University of Bristol, Bristol BS8 1TD, UK; b Bristol Medical School, MRC Integrative Epidemiology Unit, University of Bristol, Bristol BS8 2BN, UK; c Population Health Science, Bristol Medical School, University of Bristol, Bristol BS8 1UD, UK

**Keywords:** Skin, Wound healing, Scarring, GWAS, Zebrafish

## Abstract

Skin wound healing is a complex process that requires the orchestrated response of several different cell types to repair the damaged tissue and restore function. Superficial skin wounds tend to heal within days. However, larger and deeper wounds such as those caused by trauma or surgery generally heal by leaving a scar, which can impact tissue function. Scars can be debilitating, painful, and can significantly impair function. New developments to prevent and treat scarring require a deeper understanding of the cellular and molecular mechanisms associated with wound healing and scarring. Most of our current understanding of the genetics of wound healing comes from studies in animal models. However, population health approaches combined with experimental validation in animal models offer new opportunities to harness natural variability in wound repair outcomes in the population and identify new relevant biology controlling different aspects of repair and scarring in a different way. These approaches have the potential to reveal association between genetic loci and wound phenotypes in humans. Complementary experimental studies in animal models can help to validate these candidate genes and our understanding of the underlying mechanisms. In this non-systematic review, we propose the application of strategies using population health/genetics together with zebrafish models of wounding to specifically study skin scarring. We discuss potential pitfalls and strengths of the combined and complementary use of population health approaches and animal models for the identification and validation of new genes involved in skin repair, and in particular, scarring.

Any damage to the skin leads to a wound healing response. This is an orchestrated process that restores the integrity and function of the tissue. Wound healing is a tightly regulated collection of cell behaviours that proceeds through a series of overlapping phases.^1^ Over time, most adult wounds heal, but leave behind a scar, which is a stiffened tissue rich in heavily cross-linked bundles of collagen fibres.^2^

Scars can vary depending on the depth and extent of the injury, ranging from normal flat and pale scars to excessive scars, such as hypertrophic scars and keloids. Hypertrophic scars usually occur in anatomical areas of high tension and are usually red, itchy, and elevated. In contrast, proliferation in keloid lesions extends over the edges of the initial wound.^2^ Interestingly, keloid scars are more prevalent in people with darker skin,^3^ suggesting a genetic component.^2^

Given the utility of genetic associations related to phenotypes to understand the underlying genetic mechanisms of a phenotype without *a priori* assumptions, it would appear natural to exploit this in studies bridging human and experimental models. Previously, pedigree analyses have established an autosomal dominant inheritance for keloids, characterised by incomplete clinical penetrance.^4^ Genome-wide linkage studies, aimed to identify genetic susceptibility loci for a phenotype in an unbiased manner, pinpointed several regions for susceptibility to keloids, linked to genes such as *TNFAIP6* in Japanese^5^ and *EGFR* in African Americans.^5^ In contrast, candidate gene studies have explored the influence of common single nucleotide polymorphisms in genes, such as *TP53*^6^ and *TGFB1*,^7^ among others, on the susceptibility to keloid disease and hypertrophic scarring in diverse populations. Genome-wide association studies (GWAS), which do not rely on *a priori* assumptions regarding genetic mechanism, have been used to propose several susceptibility genes for keloids and hypertrophic scars.^8–14^

In this non-systematic review, we discuss the potential opportunities of using population health approaches, such as GWAS, to identify new loci associated with aspects of tissue repair, particularly scarring. We then discuss the use of zebrafish as an animal model of wound healing, and the related experimental tools to study wound healing. We finish by proposing an integrated approach, that combines unbiased discovery of genomic loci associated with scarring outcomes, with functional validation of candidate genes using *in vivo* experiments in zebrafish models of wound healing that might allow the identification of novel genes involved in driving scarring.

## GWAS as a potential tool to uncover new genes involved in scarring

### GWA studies of scarring

In the last 15 years, among the numerous GWAS (summarised in Box 1) exploring the genetic influence on fibrosis traits, a few studies have investigated scarring by focusing on keloids and post-burn hypertrophic scars.^8–16^ In these studies (summarised in Table 1 and fully detailed in ST1), the best supported association was found between the gene *NEDD4* and risk of keloids, which has been replicated across different ethnicities^8–10,14^ and confirmed in recent metanalyses of susceptibility to keloids and hypertrophic scarring (Table 1 and ST1).^16^ *NEDD4* is the founding member of the NEDD4 family of HECT (Homologous to E6AP C-terminus) ubiquitin ligases, that function in the ubiquitin proteasome system and regulate several membrane receptors, components of the endocytic machinery, and the tumour suppressor PTEN.^17^ *NEDD4* is highly expressed in cultured fibroblasts,^18^ and a study comparing normal skin samples and keloids from patients, shows higher expression of a specific transcript variant of *NEDD4* in keloids. Knock down experiments *in vivo* show that NEDD4 is involved in the activation of STAT3 and NF-κB.^19^

A study regarding risk of keloid scarring in African Americans found evidence that indicated associations of keloid susceptibility with 2 myosin genes: *MYO1E* (myosin 1E) and *MYO7A*.^8^ Further analysis including exome genotyping data revealed a rare variant in the *HYPK* gene (Huntingtin Interacting Protein K) with a possible association with keloid formation.^15^

Another gene suggested in a Japanese population from BioBank Japan^9,10^ similar Europeans from FinnGen as a marker for keloid formation is *PHLDA3* (Pleckstrin Homology Like Domain Family A Member 3). The top variants of *PHLDA3* differ between ethnicities: rs192314256 in East Asians^9,10^ and rs35383942 in Europeans.^26^ *PHLDA3*-rs35383942 is a credible variant for hypertrophic scar in the FinnGen study, along with *NEDD4, AP4E1* and *ZNF385D* genes, among others.^26^ However, none of these genes have been replicated in the UK BioBank, the other largest study among Europeans. This is also the case for associations of post-burn severity features with several genes, such as *CSMD1*,^12^ *PTPN5*,^13^ *KCNIP4* and *GALNT2*,^11^ identified in European populations, but none of these has been confirmed in follow-up analyses. Another marker that failed replication in Europeans, despite displaying strong evidence in East Asians is *FOXL2*, encoding a fork-head box (FOX) transcription factor, originally identified in a GWAS^14^ and confirmed in a meta-analysis of patients with keloids from 5 Asian populations.^27^

### Challenges of GWAS in scarring research

Population-based studies of wound healing face specific methodological challenges derived from the complexity and variability of wounds and wound management. Importantly, scars are often the result of inherently heterogeneous injuries. Additionally, heterogeneity in patient demographics, wound aetiology, response to treatments and comorbidities make it difficult to generalise findings across different populations.^28^ There are 2 main challenges to carry out GWAS on scarring: first, the lack of standardised data collection methods, which leads to inconsistencies in documentation and classification of wounds^28,29^ and second, the heterogeneity of scars, that makes it difficult to find reliable outcomes that can be compared across the population.

### Methods to assess scarring

An ideal method to measure scars would be non-invasive, accurate, reproducible and simple to implement, thus enabling the unbiased collection of clinically relevant data for research.^30^ Scarring assessment usually involves observation by clinicians, researchers or patients, which results in considerable variability among studies in how the scars are assessed and also how phenotypes are ultimately operationalised for analysis.^29^ The most popular procedures are scar rating scales, the Vancouver Scar Scale (VSS),^31^ Patient Observer Scar Rating Scale (POSAS)^32,33^ and Brisbane Burn Scar Impact Profile (BBSIP).^34^ The VSS is an objective score originally designed to describe pathologic burn scars, that assesses vascularity, scar height, pliability and pigmentation.^31^ In contrast, the POSAS consists of 2 numeric scales, one completed by the patient and another filled out by the observer.^35^ POSAS includes 16 items, covering aspects such as pain, itching, colour, stiffness, thickness and irregularity. There is also a Linear Scar version of POSAS to assess linear scars, with an extra measure item that scores the widening of scar margins.^32,33^ POSAS is known to be more consistent than VSS over repeated measures.^36^ Finally, the BBSIP is a multidimensional measure of outcomes of burns scarring based on reports from the patients.^34^

Additionally, there are objective instruments to measure the physical properties of scars, such as pliability, firmness, colour, perfusion, thickness and 3-dimensional topography.^30^ Most of these tools are known to correlate with or even outperform subjective scores.^35^ Although this specialised equipment has been shown to reliably measure physical properties of scars, it usually measures only 1 aspect of scars, its operation requires training and standardisation, making it impractical in certain clinical settings.^30^

### Phenotypic heterogeneity of scars

Keloids and hypertrophic scars are known to display significant inter- and intralesional heterogeneity.^2^ A few experimental models of scarring in humans have been developed, mostly based on surgical wounds.^37^ For example, before undergoing abdominoplasty, patients receive incisions in the lower abdomen in tissue that will later be removed.^38^ In contrast, Dunkin and colleagues developed a device to inflict highly standardised and reproducible wounds,^39^ circumventing the limitations of other models based on surgical technique.^37^ Although not practical to carry out population studies, these models could prove useful for validation and testing therapeutics.

One of the major challenges in studying scarring in humans is finding a standardised lesion that can be followed up in later life and that can be reliably compared across the population. One approach to this type of challenge is to take advantage of natural experiments, where large numbers of the population have a similar lesion. To this end, some surgical procedures and other medical interventions, such as vaccination, could provide scenarios where large numbers of people have similar lesions created by a trained professional in a standardised way.

**Figure 1:**
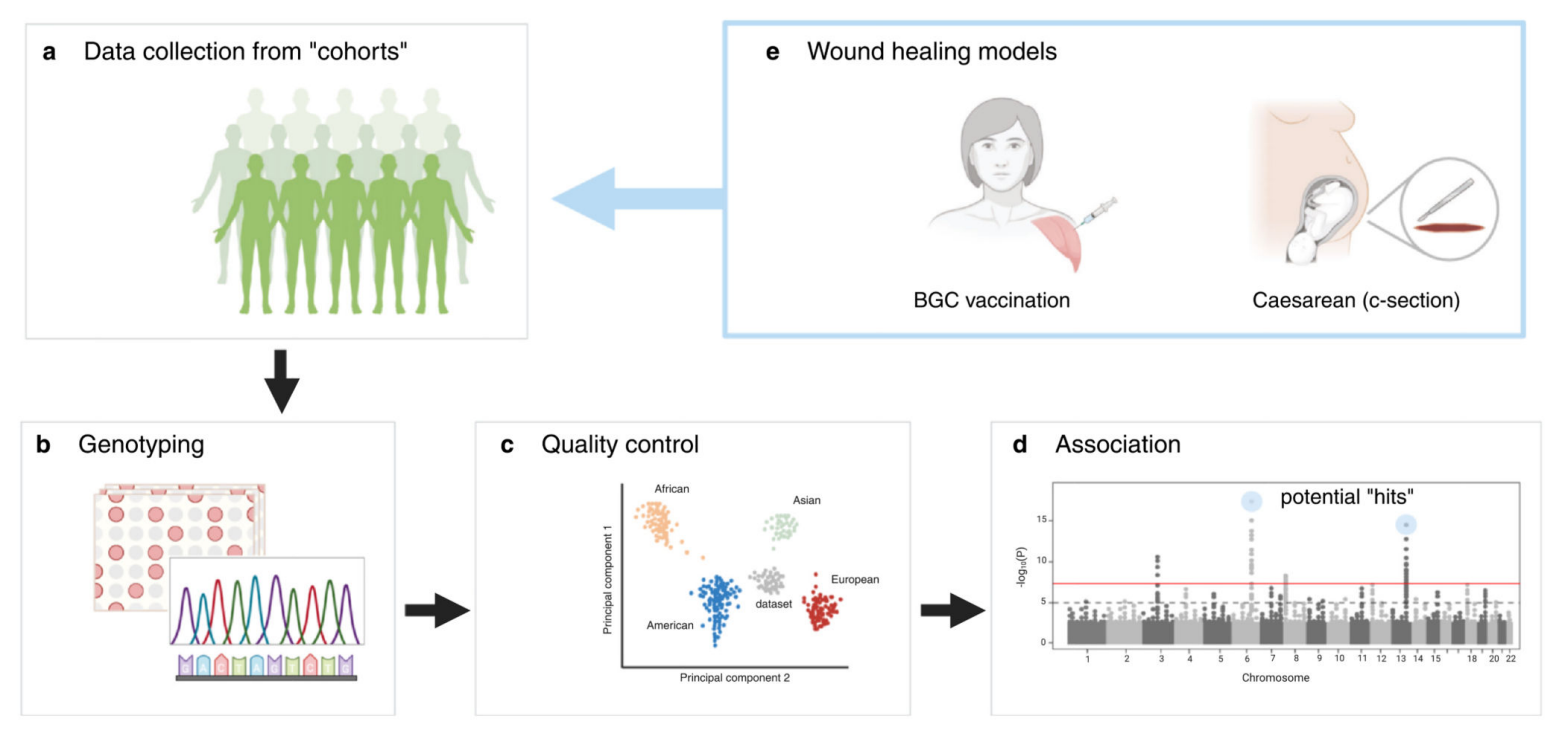
Schematic depicting GWAS workflow. (a) Data collection from study cohorts or available repositories and biobanks. (b) Genotyping using microarrays, or next generation sequencing. (c) Genetic-based clustering of the individuals as part of quality control. (d) Genetic association testing of each variant. A Manhattan plot showing the significance of the association of each genetic variant with the phenotype studied. (e) Data collection of wound healing models such as scars from BCG vaccination and C-sections, would allow population-wide study of the genetics of skin repair and scarring. Created in BioRender. Peña cabello, O. (2025) https://BioRender.com/deko0h6.

Two distinct wounds serve as good examples of this and potential models for scar research: the Bacille Calmette-Guérin (BCG) vaccine scar, a small and distinctive mark developed after administration of the BCG vaccine,^40^ and the Caesarean section scar, resulting from a C-section delivery (Figure 1e). Vaccination procedures are standardised, creating a consistent initial wound and scar formation occurs in a predictable timeframe, typically over several weeks to months.^41^ The resulting scar is generally small and localised, making it easy to observe and measure.^41^ Moreover, a vaccination ‘scar’ has some flaws as a wound scar in that it triggers an immune response beyond that of a typical wound. The surgical technique for C-sections is relatively standardised, especially for elective procedures. The location, size, and depth of the incision are fairly consistent across patients.^42^ This provides relative consistency across subjects and facilitates more direct comparisons of healing outcomes. Both scars offer opportunities for long-term wound healing studies, as they remain visible for several years, allowing for extended follow-up and measurement. Both also represent common procedures, making them suitable for population-level studies. The BCG vaccine is widely administered in over 150 countries worldwide, providing a large study pool.^43^ Worldwide, 21.1% of live births corresponded to caesareans, but regionally C-section rate can be as high as 42.8%, in Latin America and the Caribbean,^44^ offering opportunities for study pools large enough for GWAS. Findings from the investigation of the C-section wound have the additional advantage of real-world applicability, as they can be directly applied to improve patient care and surgical outcomes.

## Use of animal models for functional validation of candidate genes

If we are to use population-scale scarring events to identify genetic variants associated with aspects of the tissue repair process, then we need to consider how to validate ‘hits’ and assess whether association relates to relevant biology. This is a substantial task in almost all areas of human genetic analysis. Where possible, associations need to be validated in experimental *in vivo* models of wound healing by functional genomics studies.^21^

A wide variety of animal models have been used to study scarring,^37^ and in principle, candidate gene validation could be performed on any of these models. First, we briefly discuss the main mammalian models of wound repair and scarring, and their feasibility for large screenings in terms of cost, genetic tractability and ethical concerns. Next, we introduce the zebrafish model, discussing its genetic tractability and key advances in wound healing research. We finish discussing the advantages of zebrafish to validate large numbers of genetic associations in wound repair and scarring.

### Mammalian models of scarring

Among the mammalian models of scarring, mice are the most popular for genetic studies, and for pre-clinical studies, red Duroc pigs are favoured.^37^ The skin of pigs shares the most anatomical and physiological similarities to human skin, such as thicker epidermis, elastic dermis and hair instead of fur. Upon wounding, skin from red Duroc pigs produces a scar tissue that resembles that of hypertrophic human scars in several aspects, such as the presence of collagen bundles, dyspigmentation, hypervascularity and skin hardening.^45^ Different wounding methods have been used in red Duroc pigs to produce hypertrophic-like scars. Wounds inflicted with an electric dermatome^45^ and sharp excision wounds^46^ induce hypertrophic scarring in red Duroc pigs. The dermatome provides the advantage of fixed depth setting, helping create controlled injuries of partial thickness.^45^ Thermal injury methods have been used to elicit hypertrophic scarring. In the first model, a container with hot water is pressed against the skin of the pig, creating a contact burn.^47^ Alternatively, a contact burn can be produced using a branding iron.^48^ The hot water technique requires less skill and equipment, but the depth of the wound is difficult to control. In contrast, the temperature of the branding iron can be kept constant throughout.^48^ The similarities of scarring in the porcine models with human scarring in terms of anatomy and physiology make them the most suitable model, but pigs are big compared to other animal models, and maintenance costs together with limited genetic tractability, and ethical concerns limit their use in research.^37^

Mice and rats possess a distinctive fibromuscular layer under the skin, the panniculus carnosus, that allows the skin to glide loosely over the tissue underneath. This makes their wound healing process predominantly dependent on contraction, instead of forming large amounts of granulation tissue. A wide variety of experimental models of scarring have been developed in mice. Among them, there are models involving incision and stretch, thermal injury and several models involving the transplantation into immunosuppressed mice of human skin, human keloid scar tissue or implantation of human scar cells.^37^ However, the immune response plays a crucial role in the process of wound healing,^1^ which limits the conclusions of these studies. For example, Momtazi et al. in 2013 reported a model in which normal human skin (discarded after abdominoplasty) was transplanted onto the dorsum of immunosuppressed mice.^49^ Wounding of this skin led to scars with loss of hair and collagen arrangements consistent with hypertrophic scars. Other groups have implanted diverse cultured human cells taken from keloid tissues into immunosuppressed mice.^50,51^ These models resulted in scars with different combinations of features resembling human hypertrophic scars.^37^ Similarly, other models involve transplantation of full thickness scar tissue into immunosuppressed mice, and have been used to assess the effects of agents to decrease scarring.^52,53^ Other murine models involve incision and stretch,^54^ or punch biopsy wounds.^55^ Although mice lack the anatomical and physiological similarities to human skin and scarring that Duroc pigs have, mice models offer several advantages including their small size and reduced costs compared to those of studies using Duroc pigs, but most significantly they offer genetic tractability and a range of diverse research tools,^37^ such as reporters and knock-out transgenic strains.

### Zebrafish

#### Zebrafish as a model for reverse genetics

Zebrafish (*Danio rerio*) are tropical, freshwater fish native to the Ganges that offer several advantages for rapid reverse genetic screening. Adult zebrafish are small (2–4 cm), easy to handle, with low maintenance costs and they produce large numbers of larvae for high-throughput experiments. Importantly, zebrafish larvae develop externally and are translucent, enabling high resolution *in vivo* imaging of developmental and pathological processes.^56^

**Figure 2:**
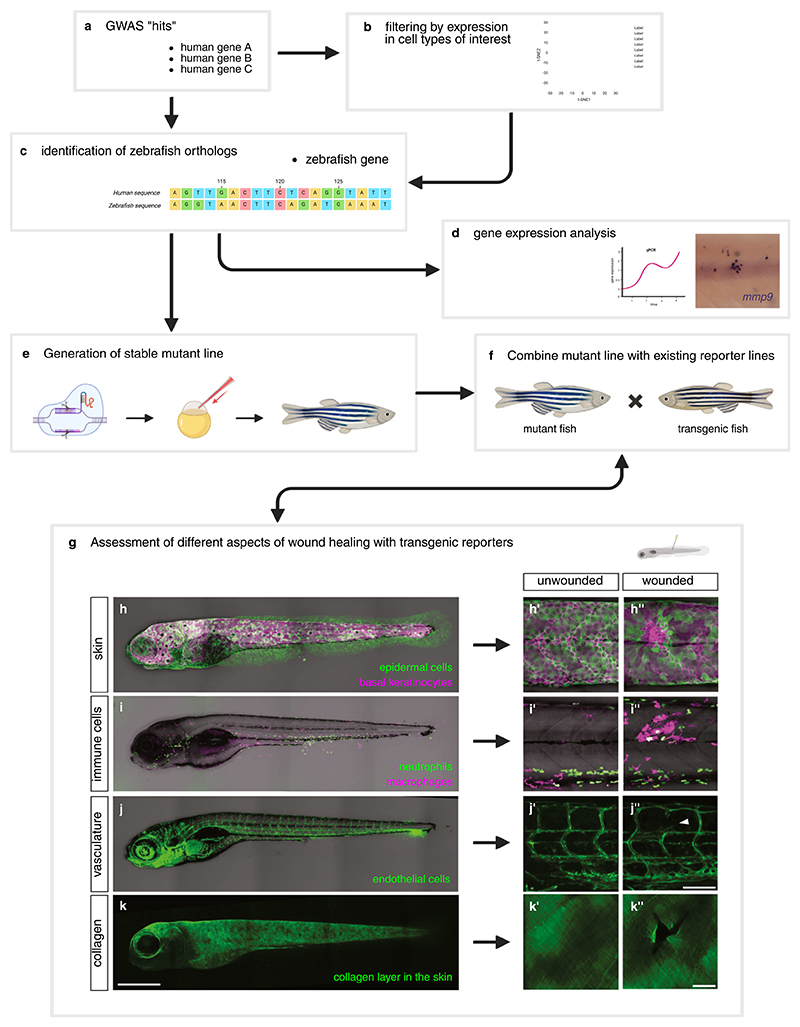
Schematic of a workflow to validate and characterise ‘hits’ from GWAS using zebrafish. (a) Candidate human genes obtained from loci with significant association in GWAS. (b) Gene list can be narrowed down by filtering based on expression in cell types of interest, using existing sc-RNAseq datasets. (c) Identification of zebrafish ortholog of the human gene of interest. (d) Gene expression analysis using qPCR (left), or *in situ* hybridisation (right). In the photo, induction of expression of *mmp9* in the midline 6 h after wounding. (e) Generation in parallel of stable mutant line of the gene of interest using the CRISPR/Cas9 system. (f) Stable mutant line for the gene of interest is combined with existing transgenic reporter fish lines. (g) Assessment of different aspects of wound healing in mutant lines combined with transgenic reporters. (h–k) Whole-mount confocal images of double transgenic larvae *krt4:GFP; krt19:tdTomato-CAAX* with labelled skin cells (h); *mpx:GFP; mfap4:tdTomato* with labelled immune cells (i); *fli1:eGFP* with labelled endothelial cells (j); and *krt19:Zcol1a2-GFP* with labelled collagen (k). Close up images of the flank of unwounded (h’–k’) and 1 day post-wounding larvae (h”–k”) showing wound closure (h”), immune cell recruitment (i”), and damage to blood vessel (j”, arrowhead) and collagen layer (k”). Scale bar in (h, i, j, and k) 500 μm, in (h’, h”, i’, i”, j’, and j”) 100 μm, and in (k’ and k”) 20 μm. Created in BioRender. Peña cabello, O. (2025) https://BioRender.com/hwwq7xt.

Established methods for transgenesis, overexpression, knockdown and genome editing^57^ allow a wide variety of applications, including the creation of several transgenic reporter lines that label specific cell types or tissues, or designed to serve as reporters of gene expression or pathway activation.^58^ Transgenic lines, in combination with the transparency of zebrafish larvae, allow detailed *in vivo* imaging of cellular processes and cell tracking. The zebrafish genome also allows the introduction of mutations by diverse techniques, with CRISPR/Cas9 system (clustered regulatory interspaced short palindromic repeats), being currently the major tool for to generate stable mutant lines in zebrafish^57^ (Figure 2e). Current CRISPR/Cas9 tools allows rapid phenotype screening in F0 animals.^59^ Similarly, tools for simultaneous disruption of multiple genes facilitate epistatic analysis, and combinatorial studies to reveal genetic pathways.^59^

#### Wound healing in zebrafish

Zebrafish have been used extensively in the field of wound healing and regeneration for several years, and numerous models have been developed to study skin wounding,^60^ cardiac^61^ and vascular^62^ injury and liver injury.^63^ Wound healing and the associated inflammatory response have been studied in zebrafish larvae and adults. Studies in adult zebrafish showed that re-epithelialisation occurs, similar to mammalian wound repair, through integrin-dependent migration, although in fish there is no initial lag phase, making the process faster than that in mammals^60^ (Figure 2h). The imaging capabilities of zebrafish have been especially advantageous to study the early events after wounding. For example, the generation and propagation of a tissue-scale gradient of the damage signal H_2_O_2_ was first shown in zebrafish by live imaging of a transgenic sensor in wounded larvae.^64^ The availability of transgenic fluorescent reporters of neutrophils and macrophages has enabled the characterisation of signals and mechanisms of recruitment and resolution in the context of skin wounds^65^ (Figure 2i) and in interaction with infections.^66^ Cell tracking and transgenic reporters have allowed the characterisation of swarming behaviour of neutrophils to wounds, and later NETosis at the wound site.^67^ Similarly, transgenic reporters labelling skin epithelial cells have been used to uncover the contribution of different cell lineages to wound re-epithelialisation in adult fish.^68^ Importantly, live imaging of transgenic reporter fish lines labelling vasculature and myeloid cells, revealed how macrophages drive neoangiogenesis at the wound site (Figure 2j) by direct interactions with wound blood vessels, and by driving the resolution of anti-angiogenic neutrophils.^62^

Adult zebrafish and larvae have remarkable regenerative capabilities, and their wounds heal without leaving scars. However, the formation of transient fibrotic scar tissue can be observed after heart injury^61^ and in skin wounds.^69^ A transgenic zebrafish line designed to label collagen fibres deposited by basal keratinocytes of the skin showed that after wounding collagen fibres are first deposited in random orientations and gradually these acquire their normal orthogonal organisation through progressive remodelling^69^ (Figure 2k).

#### Candidate gene validation using zebrafish

Given its genetic tractability and amenability for live imaging, zebrafish have been used in the past to study genes associated with heart disease,^70^ liver function^63^ and bone mineral density.^71^ A proposed pipeline to validate candidate wound genes using zebrafish is depicted in Figure 2. Once GWAS have highlighted human genes (Box 2) associated with scarring (Figure 2a), these can be narrowed down (Figure 2b), and the zebrafish orthologs identified (Figure 2c). The zebrafish and human genomes have a high conservation of coding sequences, with approximately 71% of human genes having 1 or more zebrafish orthologs.^72^ Once a zebrafish gene has been chosen for validation (Box 2), expression analysis (Figure 2d) in wounded larvae can be carried out by whole-mount *in situ* hybridisation, immunofluorescence, or by generating reporter lines,^58^ or of protein localisation, using CRISPR/Cas9-based knock-in methods.^73^ Next in the pipeline (Figure 2e), the generation of mutant lines allows functional studies of the gene of interest in wound healing. Stable germinal mutants can be achieved consistently using the CRISPR/Cas9 system in as little as 6 months.^59^ The generated mutant lines can then be used in combination with various reporter lines (Figure 2g) labelling different cell types (*e*.*g*., inflammatory cell lineages), or with other reporters relevant to wound healing, including of gene expression, pathway activation, molecule sensor, and to visualise the ECM (*e*.*g*., collagen reporter, Figure 2k). *In vivo* imaging of reporter transgenic larvae carrying mutations for a gene of interest allows the study of diverse aspects of wound healing, including re-epithelialisation (Figure 2h-h”), inflammatory response (Figure 2i-i”) and collagen (Figure 2j-j”) and vasculature (Figure 2k-k”) remodelling.

Despite the several advantages of using zebrafish as a model of wound healing, genetic differences between humans and zebrafish, as well as differences in their anatomy and physiology, lead to limitations. Importantly, zebrafish wounding does not lead to persistent scars, and similar to all other animal models, zebrafish do not naturally develop keloids.

Approximately 29% of human genes lack a zebrafish ortholog, and so any GWAS wounding ‘hits’ in such genes cannot be validated in zebrafish. Additionally, owing to an event of whole-genome duplication in the common ancestor of teleost fish, several genes in the zebrafish genome are duplicated. Consequently, for approximately 24% of human genes there is a ‘one-to-several’ relationship with zebrafish orthologs, with an average of 2.28 zebrafish genes per human gene.^72^ This can also pose a challenge for candidate validation, making it necessary to generate knockouts for all paralogs.

Human and zebrafish skin also have some anatomical and physiological differences, that make some cellular processes difficult to model in zebrafish. A key difference is the lack of skin appendages, hair, sweat glands and sebaceous glands, or analogue structures in the zebrafish skin. One of the challenges of intense scarring is the lack of skin appendages in the repaired tissue, the lack of skin appendages in zebrafish skin is a flaw in the model.

Finally, the adaptive immune systems of humans and fish also present some differences, particularly in the existence of specialised populations of lymphocytes in each species. The adaptive immune system of zebrafish is not fully mature until 4–6 weeks post-fertilisation,^77^ with B cells being one of the last immune cell populations to develop by 20 days post-fertilisation.^78^ Although this precludes the possibility of studying the roles of lymphoid cells in wound healing during the larval stages of zebrafish development, it also provides the reductionist opportunity to examine wound healing without the involvement of the adaptive immune system at these larval stages.

## Integrating population studies and animal models to study scarring

Our understanding of the genetics of scarring will benefit from a multidisciplinary integrated approach. Such an approach will combine population health methods, to identify new loci associated with skin repair and scarring outcomes in an unbiased way, together with *in vivo* experiments in animal models, that enable us to validate the genetic associations found, and to define the function of the candidate genes. Zebrafish models offer the perfect balance of low cost and high-throughput capability, with the complexity of a vertebrate organism that allows genetic modification, and live imaging to validate gene function.

GWAS of scarring outcomes will allow us to locate reliable associations between genetic variants and tissue repair (Box 2). However, signals of association between specific variants and scarring are not sufficient to prove causality or to uncover functional biology for the implicated loci.^21^ For example, GWAS signals often represent tag variants correlated with functional variants by linkage disequilibrium, complicating the identification of the causal variant underpinning the association.^20^ Furthermore, the identified genetic variants are often reliable in association, but much less informative in terms of biology—that is, not being related to simply the closest genes. To this end, association signals found in intergenic regions and gene deserts are difficult to interpret and pass on to basic science as any number of local or distant loci may be involved. Nevertheless, integration with genetically tractable animal models such as zebrafish represents a key step towards the clarification of variant association signals. Combined with existing bioinformatic approaches, mapping across to experimental systems will allow us to answer, for example, whether the neighbouring genes of an intergenic variant associated with scarring participate in the wound healing process.

Modelling of complex traits that are highly polygenic, such as schizophrenia, type 2 diabetes and heart disease,^21^ has required understanding multigenic interactions in animal models. The same is likely to be true for wound healing. CRISPR-F0 screens in zebrafish allow targeting of several genes simultaneously,^59^ enabling the combinatorial study of gene-gene interactions.

Although the conservation of coding genomic regions is high between zebrafish and human genomes, the conservation of non-protein-coding sequences is substantially lower.^72^ However, advances in genome alignment approaches,^79^ availability of more genomes,^80^ and assembly of higher quality reference genomes have enabled the identification of thousands of conserved non-protein-coding elements between zebrafish human and mouse. These advances will enable the experimental validation in zebrafish of genetic associations found in the non-coding genomic regions and shed light on the function in tissue repair of the genetic variants found in the population. Furthermore, the function of non-coding variants that are not conserved can be tested by using humanised transgenic reporters in zebrafish.^81^

## Future directions

Our understanding of the cellular and molecular mechanisms of scarring could potentially benefit greatly from integrated population health and experimental approaches and these insights can contribute to improving the prevention and treatment of scars in several ways.

Functionally validated genetic drivers of scarring may provide new potential therapeutic targets for drug development. The known differences in scarring outcomes across sex, age and ethnicity, suggest a significant genetic component that population studies could harness to develop personalised medicine approaches to scar prevention and treatment.

New population health efforts that include a wide range of populations would be instrumental in finding novel loci associated with scarring outcomes. The inclusion of individuals of African, Hispanic and Asian ancestries in new population-wide studies would benefit our understanding of hypertrophic scarring and keloids, which are more likely to develop among those groups.^82^ Similarly, future studies should aim to study less common genetic variants that could uncover genetic drivers of larger effect on tissue repair outcomes.^21^ GWAS findings could potentially be used to create genetic risk scores for predicting individual propensity for poor wound healing or excessive scarring. Multi-centre studies will be crucial in achieving sufficiently large sample sizes that enable adequate statistical power in the downstream analysis. This can only be accomplished using reliable outcome measures, standardised methodologies and robust data-sharing protocols to ensure consistency and reliability.^21^ As germline DNA variants studied by GWAS most likely explain only a fraction of the observed variability in scarring outcomes in the population, future data collection initiatives would benefit from the inclusion of epigenetic, transcriptomic, proteomic and metabolomic data. These approaches have been successful to further our understanding of wound healing and scarring in experimental contexts,^83^ and in samples from patient scars,^84^ but their combination with population health approaches has been slower.^85^

The validation of potential new genes involved in tissue repair and scarring using experimental models of wound healing will benefit from recent advances in genome editing technologies, *in vivo* imaging and development of genetically encoded sensors for molecules and signalling pathways all of which can be easily applied in zebrafish studies. New applications of the CRISPR/Cas9 system allow the generation of lineage-specific blocking of gene function lines^86^ and help achieve temporal control over Cas9 activity by combining this system with optogenetic tools.^87^ These technical improvements will allow us to generate models of gene loss of function in specific cell types that can be induced at different stages of wound healing.

## Conclusion

Our understanding of tissue repair and scarring has benefitted from hypothesis driven studies using experimental models of wound healing. Population health approaches, such as GWAS, offer substantial potential for identifying new loci linked to various aspects of tissue repair and scarring. Combining such GWAS focused on wound repair and scarring phenotypes, with the systematic use of experimental *in vivo* models of wounding, will provide new insights into the genetic basis of wound healing and scarring variability in the population and will identify novel genes and pathways involved in regulating tissue repair. This unbiased approach should also enable us to soon find potential pharmacological targets to develop better therapeutics to treat pathologies of wound healing and scarring.

## Supplementary Material

**Appendix A. Supporting information**

Supplementary data associated with this article can be found in the online version at doi:10.1016/j.bjps.2025.11.010.

Supplementary Material

## Figures and Tables

**Table 1:** Summary of GWA studies on scarring phenotypes. Associations with a p-value < 5E-08 found in intragenic regions are shown.

PubMed ID	First author	Year	Phenotype	Ethnicity	Cohort	Sample size	Genomic approach	Genes	Ref.
20711176	Nakashima	2010	Keloid	East Asians	BioBank Japan	175 cases 906 controls	GWAS	*LOC12490451, NEDD4*	14
32514122	Ishigaki	2020	Keloid	East Asians	BioBank Japan	812 cases 211,641 controls	GWAS	*AL356108.1, BPESC1, NEDD4, PHLDA3*	10
34594039	Sakaue	2021	Keloid	East Asians	BioBank Japan	1055 cases 177,671 controls	GWAS	*AL356108.1, BPESC1, NEDD4, PHLDA3*	9
				Europeans East Asians	BioBank Japan + UK BioBank + FinnGen	668 European cases 481,244 European controls	META-GWAS		
						1055 East Asian cases			
						177,671 East Asian controls			
https://metaresults- ukbb.finngen.fi/	Kurki	2023	Hypertrophic scar	Europeans	FinnGen R12 + UK BioBank	2357 cases 806,484 controls	META-GWAS	*AP4E1, FBN1, LINC01226, MYO5A, NEDD4, PHLDA3, USP8, WDR72, ZNF385D*	25
https://mvp-ukbb. finngen.fi/					FinnGen R12 + UK BioBank + Million Veteran Program	6845 cases 1,375,914 controls	META-GWAS	*AP4E1, BABAM2, FBN1, GALK2, LINC01226, LSP1, MYO5A, NEDD4, PHLDA3, RP4-734C18.1, SETBP1, SLC22A18AS, TAB2, USP8, WDR72*	
https://r12.finngen.fi/					FinnGen R12	2068 cases 465,673 controls	GWAS	*AP4E1, FBN1, MYO5A, PHLDA3, USP8, WDR72, ZNF385D*	
39746571	Dand	2025	Keloid/ Hypertrophic scar	Europeans	FinGenn	934 cases 224,189 controls	GWAS	*PHLDA3*	16
					FinnGen + UK BioBank	1777 cases 412,002 controls	META-GWAS	*PHLDA3*	

## Box 1 Genome-wide association studies (GWAS)

In the last 2 decades, GWAS have proven to be a powerful approach in uncovering the genetic basis of complex traits, assessing disease risk prediction and contributing to tailored prevention and treatment strategies,^20^ and in drug discovery, providing information for drug development and repurposing strategies.^20,21^ GWAS typically involve the examination of single nucleotide polymorphism/variants (SNP or SNV), across the genome to identify genetic associations with phenotypes and diseases of interest (Figure 1). Depending on sample size and data performance, this approach usually focuses on the analysis of common variants (with frequencies greater than 1%) (Figure 1a-c). Each variant is individually tested for its association with the phenotype of interest (Figure 1d) and the resulting effect estimates and p-values are then used to summarise which of those variants show the strongest associations to any given phenotype. In this context, statistical power is an important consideration. Power is contingent on the frequency of variation, effect sizes, sample size and stringency of evidence required, and is routinely improved through the inclusion of thousands to millions of participants in individual or meta-analysed efforts.^21^ The potential functional roles of flagged variants and of the genes in their vicinity are not known through GWAS, but are reliably associated with the outcomes. Importantly, these flags are signposts for further exploration in post-GWAS studies, including *in silico* and functional analyses as we outline in this article.

GWAS provide an approach to find genetic associations without *a priori* assumptions about genetic mechanism, and can be used to discover new genes and pathways that are not known to be involved in the studied trait.^21^ However, common variants typically account for only a small portion of the total heritability estimated through other methods.^21,22^ Additional factors contributing to this discrepancy include structural variation, genetic architecture and non-additive effects, driven by epistatic or gene-environment interactions.^22^ GWAS is limited to the detection of germline variants. Alternatives to GWAS, such as whole-exome sequencing studies (WES), can capture rare variants associated with a phenotype, but they are limited to the protein-coding regions of the genome.^23^ In contrast, whole-genome sequencing studies (WGS) can measure nearly all genetic variation in the human genome and assess structural variants, but their high cost limits their use, and they are constrained to the phenotypes available in large-scale cohort resources, such as the UK Biobank.^24^

## Box 2 From variants in the population to genes in model organisms

A critical element of the pipeline proposed here is the transition from the identification of genetic variants found in the human population associated with scarring, to the study of gene function in experimental wound healing in zebrafish. This transition is made in 2 steps: first, the genetic variants associated with the scarring phenotype help pinpoint a human gene; and next, an orthologous gene in the model organism is found for that human gene.

GWAS result in the identification of genetic variants associated with the studied phenotype across the genome. Leaving aside particular situations, for example, where associated variants fall into coding sequences, moving from those single nucleotide polymorphism (SNP) signals to a gene is extremely complex. Fine mapping can be used to identify likely causal variants, or parallel reporter assays can be used to assess the ability of each variant to drive gene expression or other outcome. GWAS data can also be integrated with functional annotation of the genome to help identify regulatory sequences that could be perturbed by the associated variant.^21^ Importantly, unequivocal identification of one gene is unnecessary, as the critical advantage of this strategy lies in identifying candidate genes with a possible role in scarring, which can be later tested experimentally in downstream steps of the pipeline.

Next, to study gene function using a model organism requires the identification of an orthologous gene in the model organism. The new tools and resources currently available make the routine identification of ortholog genes in model organisms relatively fast and simple. Sites such as https://www.ensembl.org/^74^ have pre-made sequence alignments of genomic regions, cDNA and protein sequences, to assess synteny, and similarity of ortholog genes. Other resources, such as https://www.uniprot.org,^75^ using recent advances in protein structure prediction, such as https://www.alphafold.ebi.ac.uk,^76^ provide additional support for orthology relationships. In any model organism, this step is limited by the conservation of coding sequences, as well as other factors, such as local or total genome duplication, as discussed for zebrafish^72^ (see main text).
